# Amikacin toxicity revisited: pentoxifylline offers protection in high-risk treatment scenarios

**DOI:** 10.1186/s13568-025-01949-8

**Published:** 2025-10-22

**Authors:** Nada Moustafa, Mona B. Abd El-latif, Alyaa Farid

**Affiliations:** 1 Biotechnology Department, Faculty of Science, Cairo University, Giza, Egypt; 2 Environmental Research Department, Theodor Bilharz Research Institute, Giza, Egypt

**Keywords:** Amikacin, Pentoxifylline, Oxidative stress, Inflammation, Kidney, Liver

## Abstract

Amikacin (AMK), a potent aminoglycoside antibiotic, is clinically valuable for severe Gram-negative infections but is limited by its nephrotoxic and hepatotoxic effects, primarily mediated through oxidative stress and inflammation. This study investigated the protective role of pentoxifylline (PTX), a methylxanthine derivative with antioxidant and anti-inflammatory properties, against AMK-induced organ damage in male BALB/c mice. Thirty mice were divided into six groups: control, AMK (100 mg/kg/day), PTX monotherapy (50 or 100 mg/kg/day), and AMK combined with PTX (50 or 100 mg/kg/day). After 28 days, biochemical, oxidative stress, inflammatory, and histopathological analyses were conducted. AMK administration significantly elevated renal (BUN and creatinine) and hepatic (ALT, AST and ALP) markers, increased oxidative stress (MDA), and upregulated inflammatory cytokines (IL-17), alongside histopathological damage in kidney and liver tissues. Co-treatment with PTX, particularly at 100 mg/kg, normalized these parameters, restored antioxidant defenses, reduced inflammation, and preserved tissue architecture. PTX demonstrated dose-dependent efficacy, with the higher dose offering complete protection against AMK-induced toxicity. These findings highlighted PTX’s potential as an adjunctive therapy to mitigate AMK-associated nephrotoxicity and hepatotoxicity, suggesting its clinical utility in optimizing aminoglycoside safety without compromising efficacy.

## Key points

- PTX effectively prevents both nephrotoxicity and hepatotoxicity induced by AMK.
- Complete organ protection at 100 mg/kg PTX, with normalization of biochemical markers, oxidative stress, and histopathology.
- PTX is proposed as a safe, adjunctive therapy to enhance AMK safety without compromising its antimicrobial efficacy, addressing a critical need in antibiotic stewardship.

## Introduction

Aminoglycosides represent an important class of antibiotics widely employed to treat severe bacterial infections (Kotra et al. 2000; Vakulenko and Mobashery 2003). Beyond their antimicrobial role, these compounds have demonstrated therapeutic potential in genetic disorders, such as nonsense mutation suppression in hemophilia (James et al. 2005; Keeling et al. 2012), and in managing Meniere’s disease through intratympanic gentamicin administration (Pullens and van Benthem 2011). Additionally, their ability to inhibit HIV replication highlights their expanding pharmacological relevance (Schroeder et al. 2000; Houghton et al. 2010). Structurally, most aminoglycosides consist of an aminocyclitol ring (e.g., streptamine or 2-deoxystreptamine) linked to amino sugars, though exceptions like spectinomycin, an aminocyclitol lacking amino sugars, also exhibit antibiotic properties (Veyssier and Bryskier 2005). Streptomycin, the first aminoglycoside discovered in 1944, remains clinically significant (Jones et al. 1944; Davies 2006), followed by the introduction of neomycin (Waksman and Lechevalier 1949), kanamycin (Umezawa 1958), and gentamicin (Weinstein et al. 1963). Subsequent discoveries, including tobramycin, further expanded this class, with some derivatives like paromomycin showing efficacy against parasitic infections such as leishmaniasis (Davidson et al. 2009; Chandrika and Garneau-Tsodikova 2016). Natural aminoglycosides are predominantly synthesized by *Streptomyces* (suffix “-mycin”) or *Micromonospora* (“-micin”) species, underscoring their microbial origin (Tolmasky 2007; Yao and Moellering 2007). Their diverse mechanisms, from ribosomal targeting to RNA modulation, continue to inspire research into novel applications and resistance mitigation (Fosso et al. 2015; Serio et al. 2017).

Amikacin (AMK) is a semisynthetic aminoglycoside antibiotic derived from kanamycin A via acylation of the 1-amino group of the 2-deoxystreptamine moiety, first introduced clinically in 1972 (Ramirez and Tolmasky 2017). This broad-spectrum agent (molecular weight: 585.6 g/mol, C_22_H_43_N_5_O_13_) exhibits characteristic pharmacokinetics including a short elimination half-life (2–3 h in adults with normal renal function) and limited plasma protein binding (< 10%), facilitating extensive extracellular distribution while restricting cellular penetration (Sizar et al. 2023; Turnidge 2003). AMK’s distinctive L-hydroxy-γ-aminobutyryl side chain at position 1 renders it resistant to most aminoglycoside-modifying enzymes, making it particularly effective against resistant Gram-negative pathogens (Shaw et al. 1993). Its hydrophilic nature and low plasma protein binding (< 10%) contribute to its extensive distribution into extracellular fluids while limiting penetration into cells and tissues (Turnidge 2003).

AMK’s unique resistance to most aminoglycoside-modifying enzymes has established it as a crucial therapeutic option for treating infections resistant to other aminoglycosides, making it the most widely used semisynthetic aminoglycoside (Yu et al. 1977; Sklaver et al. 1978; Ristuccia and Cunha 1985; Gerding et al. 1991; Gad et al. 2011; Marsot et al. 2017; Pacifici and Marchini 2017). Its pharmacokinetic profile closely resembles that of natural aminoglycosides gentamicin and tobramycin, reaching peak serum concentrations within 30–60 min following intravenous administration (Radigan et al. 2010). Optimal antibacterial activity occurs when peak serum concentrations reach 8–10 times the minimal inhibitory concentration (MIC) (Radigan et al. 2010). Clinically, AMK—either as monotherapy or in combination regimens—demonstrates efficacy against serious infections caused by aerobic Gram-negative bacteria, mycobacteria, and Nocardia species (Ambrosioni et al. 2010; Caminero et al. 2010; MacDougall and Chambers 2011; Tamma et al. 2012; White et al. 2015; Yuan 2015; Marsot et al. 2017). Notably, it remains an essential therapeutic agent for life-threatening neonatal infections (Sherwin et al. 2009; Siddiqi et al. 2009; Tayman et al. 2011; Pacifici and Marchini 2017).

Aminoglycosides preferentially accumulate in proximal tubule epithelial cells, driving nephrotoxicity through selective renal concentration (Karasawa and Steyger 2011; Ehsani et al. 2017). AMK undergoes minimal metabolism and renal excretion, generating reactive oxygen species (ROS) that induce apoptotic damage (Aşçı et al. 2015). This leads to clinically significant acute kidney injury (AKI) with substantial morbidity/mortality, underscoring the need for early intervention (Ehsani et al. 2017). Clinical evidence suggests that aminoglycoside-induced liver injury is exceptionally accidental, potentially due to dose-limiting nephrotoxicity and ototoxicity that restrict cumulative drug exposure (Kalakonda et al. 2017). While isolated case reports describe idiosyncratic hepatotoxicity with some aminoglycosides, the causal relationship remains uncertain in many instances (National Institute of Diabetes and Digestive and Kidney Diseases 2019). They have been implicated in extremely rare instances of cholestatic hepatitis, typically manifesting 1–3 weeks post-initiation of therapy and frequently accompanied by systemic hypersensitivity features including rash, fever, and occasional eosinophilia (Gillies et al. 2015). These hepatic reactions uniformly demonstrate complete resolution within 1–2 months, with no reported cases of chronic liver injury (Bernal and Wendon 2013).

Emerging evidence demonstrates that AMK induces hepatotoxicity through interconnected pathological mechanisms. At the cellular level, it triggers oxidative stress (evidenced by elevated MDA and depleted GSH) and mitochondrial dysfunction, leading to characteristic histopathological changes including vacuolar degeneration, sinusoidal dilation, and mononuclear infiltration (Kadam et al. 2013; Azirak and Özgöçmen 2023; Habeeb 2023). Ultrastructural studies reveal profound subcellular damage, featuring mitochondrial cristae disruption, smooth ER hyperplasia, and microcholestasis—hallmarks of impaired detoxification (Martines et al. 1988). The toxicity cascade is amplified by AMK’s induction of CYP2B1/2 enzymes, which promote ROS generation and subsequent activation of apoptotic (caspase-3) and inflammatory (TNF-α) pathways (Azirak and Özgöçmen 2023). These findings establish AMK as a multifaceted hepatotoxin whose effects are particularly pronounced during extended or high-dose regimens, highlighting the need for protective interventions targeting oxidative and inflammatory mediators.

Pentoxifylline (PTX), a methylxanthine derivative with potent anti-inflammatory, antioxidant, and vasodilatory properties, has shown promise in mitigating drug-induced organ toxicity (Zhang et al. 2004; McCormick et al. 2008; Du et al. 2014). Studies indicated that PTX reduces oxidative stress by scavenging ROS and enhancing endogenous antioxidant enzymes, such as GSH, while simultaneously suppressing pro-inflammatory cytokines like TNF-α (Zhang et al. 2005; Garcia et al. 2015). Given that AMK toxicity is mediated by ROS generation, mitochondrial dysfunction, and apoptosis (Laurent et al. 1982; Azirak and Özgöçmen 2023), PTX could counteract these effects by improving microcirculation, reducing lipid peroxidation, and inhibiting apoptosis activation (Dhulqarnain et al. 2021; Wang et al. 2024; Alherz et al. 2025). For instance, in animal models, PTX attenuated glycoxidative stress by inhibiting advanced glycation end products (AGE) and their receptors (Inacio et al. 2020). In the liver, PTX attenuated acetaminophen-induced hepatotoxicity by reducing oxidative stress and inflammation (Abdel Salam et al. 2005). Similarly, PTX and thiamine synergistically attenuate rhabdomyolysis-induced acute kidney injury in rats through dual inhibition of TLR4/NF-κB signalling and NLRP3 inflammasome-mediated pyroptotic pathways (Al-Kharashi et al. 2023). Taye et al. (2009) reported the protective effect of PTX against D-galactosamine-induced hepatotoxicity in rat model. Mohammed et al. (2024) showed that PTX ameliorated carbamazepine-induced hepatotoxicity by down regulation of *CYP3A4* and *NF-kB* gene in rats. Its broad therapeutic potential is further supported by studies in doxorubicin cardiotoxicity (Elshazly et al. 2016) and bleomycin-induced pulmonary fibrosis (Entzian et al. 1997), where PTX mitigated inflammation and fibrosis. These findings underscore PTX’s pleiotropic mechanisms, making it a compelling candidate to counteract AMK-induced toxicity. Moreover, like the protective effects shown for other agents in cisplatin- and gentamicin-induced injury (Bazmandegan et al. 2017, 2021; Amirteimoury and Fatemi 2023), PTX may offer comparable benefits against AMK toxicity through shared antioxidant mechanisms.

Clinically, finding a protective approach that can be co-administered with AMK may protect tissues/organs without compromising its antimicrobial efficacy, particularly in high-risk patients requiring prolonged therapy. This study aimed to evaluate the potential protective effects of PTX against AMK-induced nephrotoxicity and hepatotoxicity in an experimental animal model. This was performed by assessing the renal and hepatic function, oxidative stress and inflammation. A histopathological examination was conducted in kidney and liver tissue to validate the structure preservation that resulted from PTX administration.

## Materials and methods

### Ethical approval and study design

The experimental protocol was carried out strictly in accordance with the ARRIVE principles and obtained ethical approval from Cairo University’s Institutional Animal Care and Use Committee (IACUC) (Approval No.: CUIF6723). A minimum of five mice per group was required, according to a power analysis performed with G*Power 3.1 software based on preliminary data with an assumed medium effect size (f = 0.25), α-level of 0.05, and 80% power. 30 male BALB/c mice (8–10 weeks old, 25–30 g) were obtained from the National Research Centre (Cairo, Egypt) which were pathogen-free.

### Animal housing and randomization

Mice were kept under rigorous environmental control in ventilated cages with a twelve-hours light/dark cycle, a temperature of 22 ± 2 °C, and a relative humidity of 50 ± 2%. Every animal was given unlimited access to purified water and standard rodent food. To guarantee an unbiased distribution, mice were randomly assigned into six experimental groups (*n* = 5/group) after a seven-day acclimatization period.

### Experimental groups and treatment protocol

The study comprised: (1) a saline-treated control group; (2) an AMK (Sigma-Aldrich, USA)-induced toxicity group (100 mg/kg/day i.p.); (3) two PTX (Sigma-Aldrich, USA) monotherapy groups (50 and 100 mg/kg/day orally) (Asvadi et al. 2011; Brasileiro et al. 2013); and (4) two combination therapy groups receiving AMK (100 mg/kg/day i.p.) with either dose of PTX (50 or 100 mg/kg/day orally). All treatments were administered between 10:00 to 11:00 daily for 28 consecutive days to control for circadian variations. AMK was prepared fresh daily in sterile saline (0.9% NaCl) according to established protocols (Batoo et al. 2018).

### Monitoring and sample collection

Animals were monitored daily for clinical signs including weight fluctuations, fur condition alterations, and activity level changes. Terminal procedures were performed under sodium pentobarbital anesthesia (50 mg/kg i.p.), with blood collected via cardiac puncture and centrifuged at 1500 rpm for 15 min at 4 °C to obtain serum, which was stored at − 80 °C. The kidneys and livers were either homogenized in ice-cold Tris-HCl buffer (10 mM, pH 7.4) for biochemical studies or fixed in 10% neutral buffered formalin for 24 h for histological processing.

### Quality control measures

To ensure experimental consistency: (1) all solutions were prepared fresh daily; (2) injection sites were alternated to prevent local irritation; (3) researchers were blinded to treatment groups during data collection and analysis; and (4) environmental parameters were continuously monitored. The selected PTX doses were based on previous efficacy studies demonstrating therapeutic effects without toxicity (Asvadi et al. 2011; Brasileiro et al. 2013), while the AMK regimen followed established nephrotoxicity protocols (Batoo et al. 2018).

### Kidney function assessment

Serum biomarkers of renal function were quantified using commercially available ELISA kits according to the manufacturers’ protocols. Urea levels were determined using ab83362 (Abcam, USA), creatinine with ab65340 (Abcam, USA), uric acid with ab65344 (Abcam, USA) and albumin with ab108791 (Abcam, USA). All assays were performed with appropriate controls to ensure analytical precision. Serum samples were thawed on ice and centrifuged at 10,000 × g for 5 min prior to analysis to remove potential particulates.

### Oxidative stress and inflammatory marker evaluation

Oxidative stress parameters were assessed in kidney and liver homogenates. Lipid peroxidation was evaluated by measuring malondialdehyde (MDA) levels (MBS741034, MyBioSource, USA), while antioxidant capacity was determined through superoxide dismutase (SOD, MBS2707323) and reduced glutathione (GSH, MBS267424) activities (MyBioSource, USA). The pro-inflammatory cytokine IL-17 was quantified using a high-sensitivity ELISA kit (E-EL-M0047, Elabscience, USA).

### Histopathological examination

Following euthanasia, kidney and liver tissues were carefully collected and immediately fixed in 10% neutral buffered formalin for 24–48 h at 4 °C to ensure optimal tissue preservation. The fixed tissues underwent standard processing involving dehydration through a graded ethanol series (70%, 80%, 90%, and 100%), clearing in xylene, and embedding in paraffin wax. Using a rotary microtome (Leica RM2235, Germany), 4–5 μm thick sections were obtained and mounted on slides. For comprehensive histological evaluation, tissue sections were stained with hematoxylin & eosin (H&E) for general morphological assessment.

### Statistical analysis

After confirming normality, one-way ANOVA with Tukey’s post-hoc test was applied for multiple comparisons. Results were presented as mean ± standard deviation (SD) with statistical significance set at *p* < 0.05. All randomized animals (*n* = 5/group) completed the study protocol without attrition, yielding complete datasets for analysis.

## Results

### Kidney function analysis

**Fig. 1:**
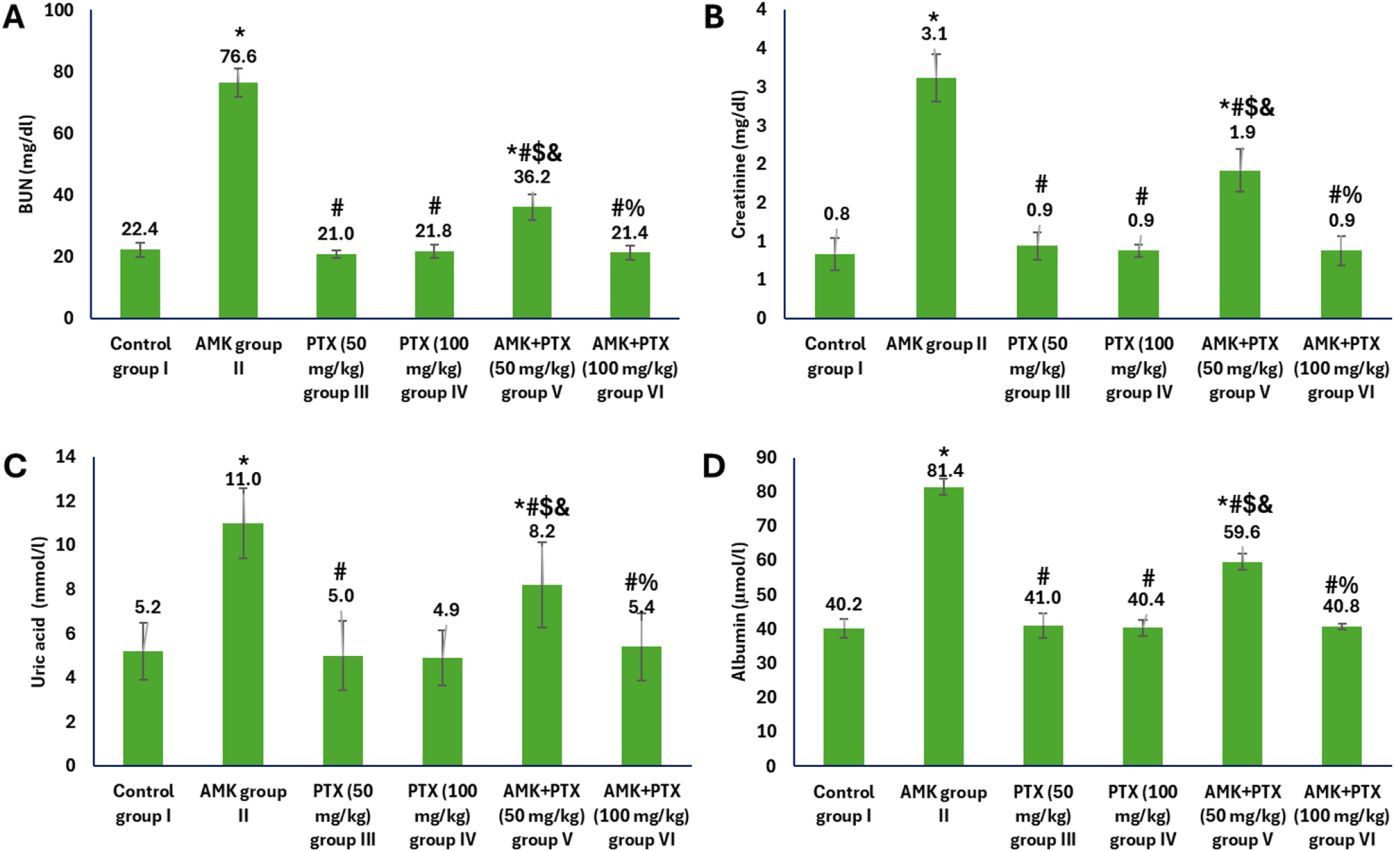
Kidney function parameters in different experimental groups showing levels of BUN (**A**), creatinine (**B**), uric acid (**C**) and albumin (**D**). ^*, #, $, & and %^ represented significance (*p* < 0.05) with respect to control group I, AMK group II, PTX (50 mg/kg) group III, PTX (100 mg/kg) group IV and AMK + PTX (50 mg/kg) group V

The biochemical analysis of renal function markers revealed significant differences among the different experimental groups (Fig. 1). In the AMK-administrated group II, marked elevations were observed in all measured parameters compared to the control group I, with BUN increasing from 22.4 to 76.6 mg/dL, creatinine from 0.8 to 3.1 mg/dL, uric acid from 5.2 to 11.0 mmol/L, and albumin from 40.2 to 81.4 µmol/L, demonstrating substantial nephrotoxicity. Both PTX monotherapy groups (III and IV) maintained values comparable to controls, indicating no adverse renal effects from PTX administration alone. Notably, co-treatment with PTX at 50 mg/kg (group V) significantly attenuated AMK-induced renal damage, reducing BUN to 36.2 mg/dL, creatinine to 1.9 mg/dL, uric acid to 8.2 mmol/L, and albumin to 59.6 µmol/L. Most remarkably, the higher dose of PTX (100 mg/kg) in combination with AMK (group VI) completely normalized all renal parameters (BUN: 21.4 mg/dL; creatinine: 0.9 mg/dL; uric acid: 5.4 mmol/L; albumin: 40.8 µmol/L), demonstrating a dose-dependent protective effect against AMK-induced nephrotoxicity.

### Liver function analysis

**Fig. 2:**
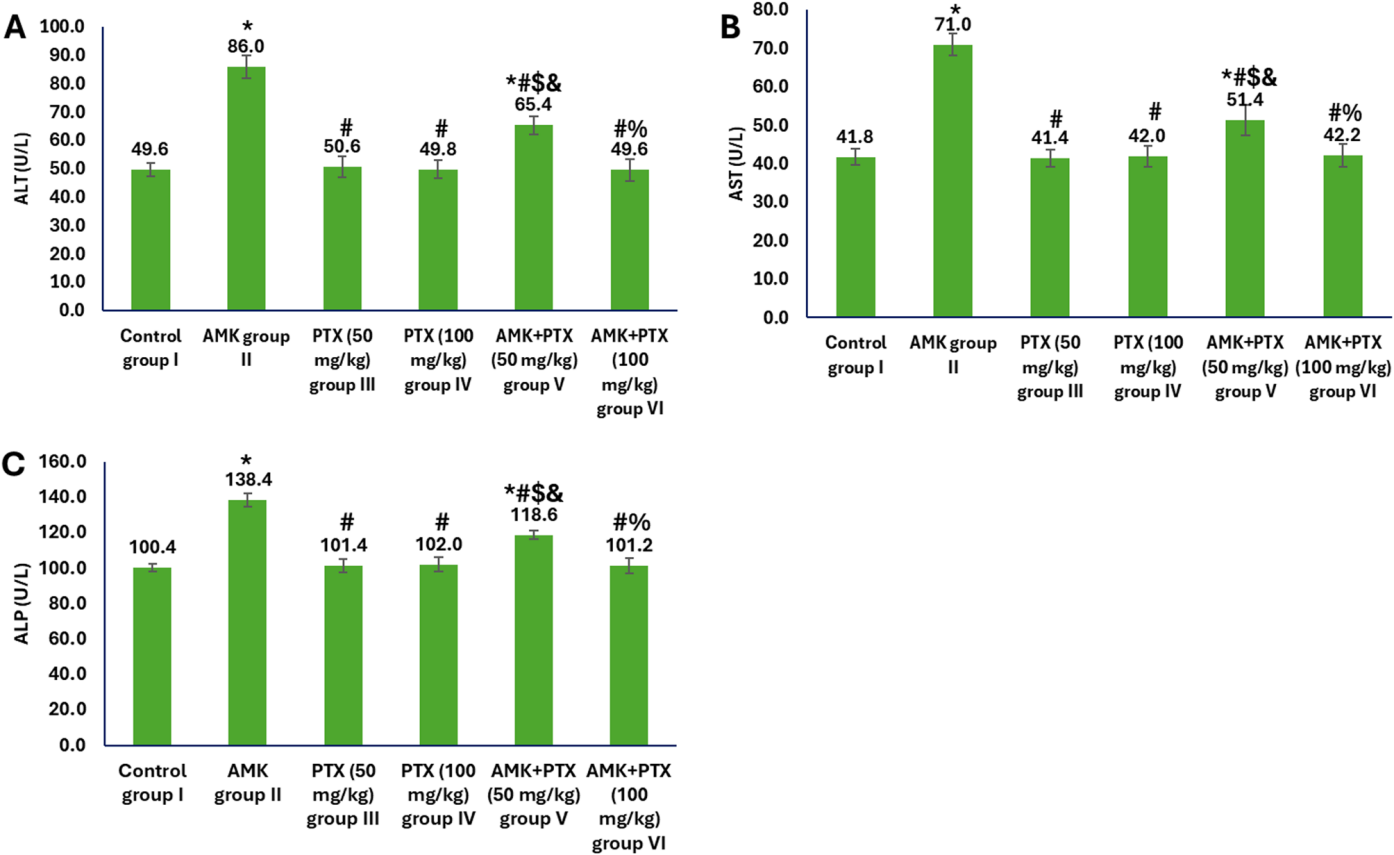
Liver function parameters in different experimental groups showing levels of ALT (**A**), AST (**B**) and ALP (**C**). ^*, #, $, & and %^ represented significance (*p* < 0.05) with respect to control group I, AMK group II, PTX (50 mg/kg) group III, PTX (100 mg/kg) group IV and AMK + PTX (50 mg/kg) group V

The evaluation of hepatic function markers demonstrated distinct patterns among the experimental groups (Fig. 2). Animals treated with AMK alone (group II) exhibited significant elevations in liver enzymes compared to the control group I, with ALT increasing from 49.6 to 86.0 U/L, AST from 41.8 to 71.0 U/L, and ALP from 100.4 to 138.4 U/L, indicating substantial hepatotoxicity. In contrast, both PTX monotherapy groups (III and IV) maintained liver enzyme levels nearly identical to controls, confirming the absence of hepatic adverse effects from PTX administration. The combination treatment with AMK and PTX at 50 mg/kg (group V) showed partial protection, significantly reducing AMK-induced enzyme elevations. Most notably, co-administration of the higher PTX dose (100 mg/kg) with AMK (group VI) completely prevented hepatic damage, maintaining all enzyme levels within normal ranges (ALT: 49.6 U/L; AST: 42.2 U/L; ALP: 101.2 U/L), which was like control values.

### Oxidative stress and inflammation analysis in kidney

**Fig. 3:**
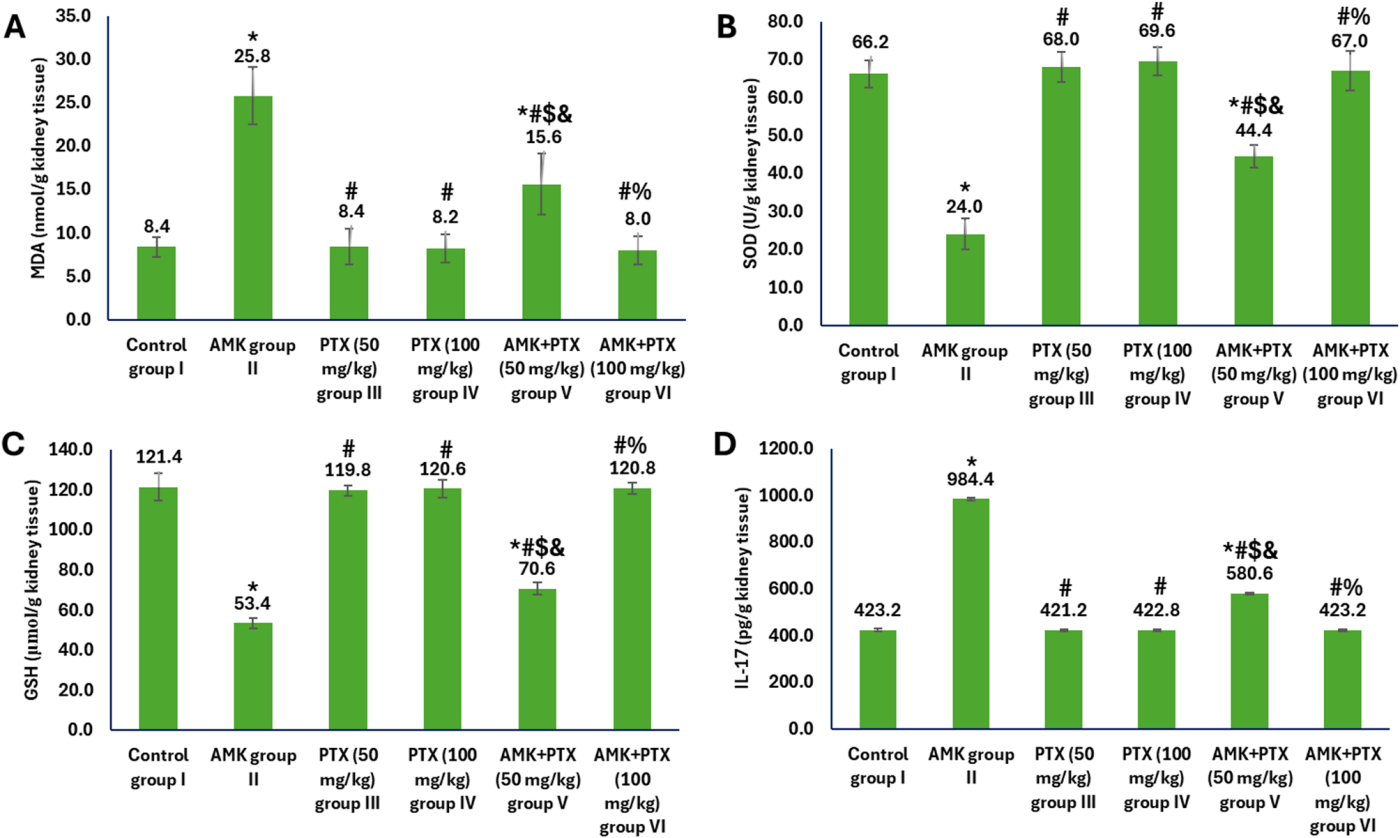
Oxidative stress and inflammatory markers in different experimental groups showing levels of MDA (**A**), SOD (**B**), GSH (**C**) and IL-17 (**D**) in kidney tissue homogenates. ^*, #, $, & and %^ represented significance (*p* < 0.05) with respect to control group I, AMK group II, PTX (50 mg/kg) group III, PTX (100 mg/kg) group IV and AMK + PTX (50 mg/kg) group V

The analysis of oxidative stress and inflammatory markers in kidney tissue revealed significant variations among the experimental groups (Fig. 3). AMK administration alone (group II) induced substantial oxidative damage, evidenced by a marked increase in MDA levels (25.8 vs. 8.4 nmol/g kidney tissue in controls group I), along with significant reductions in the antioxidant enzymes levels (SOD: 24.0 vs. control 66.2 U/g kidney tissue; GSH: 53.4 vs. control 121.4 µmol/g kidney tissue). AMK administration also triggered a pronounced inflammatory response, elevating IL-17 levels to 984.4 pg/g kidney tissue versus 423.2 pg/g kidney tissue in control group I. PTX monotherapy at both doses (groups III and IV) maintained oxidative and inflammatory markers at control levels, indicating no adverse effects of PTX administration. Co-treatment with AMK and PTX (50 mg/kg) (group V) demonstrated partial protection, significantly mitigating AMK-induced oxidative stress and inflammation. Most remarkably, the higher PTX dose (100 mg/kg) combined with AMK (group VI) completely prevented oxidative and inflammatory damage, restoring all markers to control values (MDA: 8.0 nmol/g kidney tissue; SOD: 67.0 U/g kidney tissue; GSH: 120.8 µmol/g kidney tissue; IL-17: 423.2 pg/g kidney tissue).

### Oxidative stress and inflammation analysis in liver

**Fig. 4:**
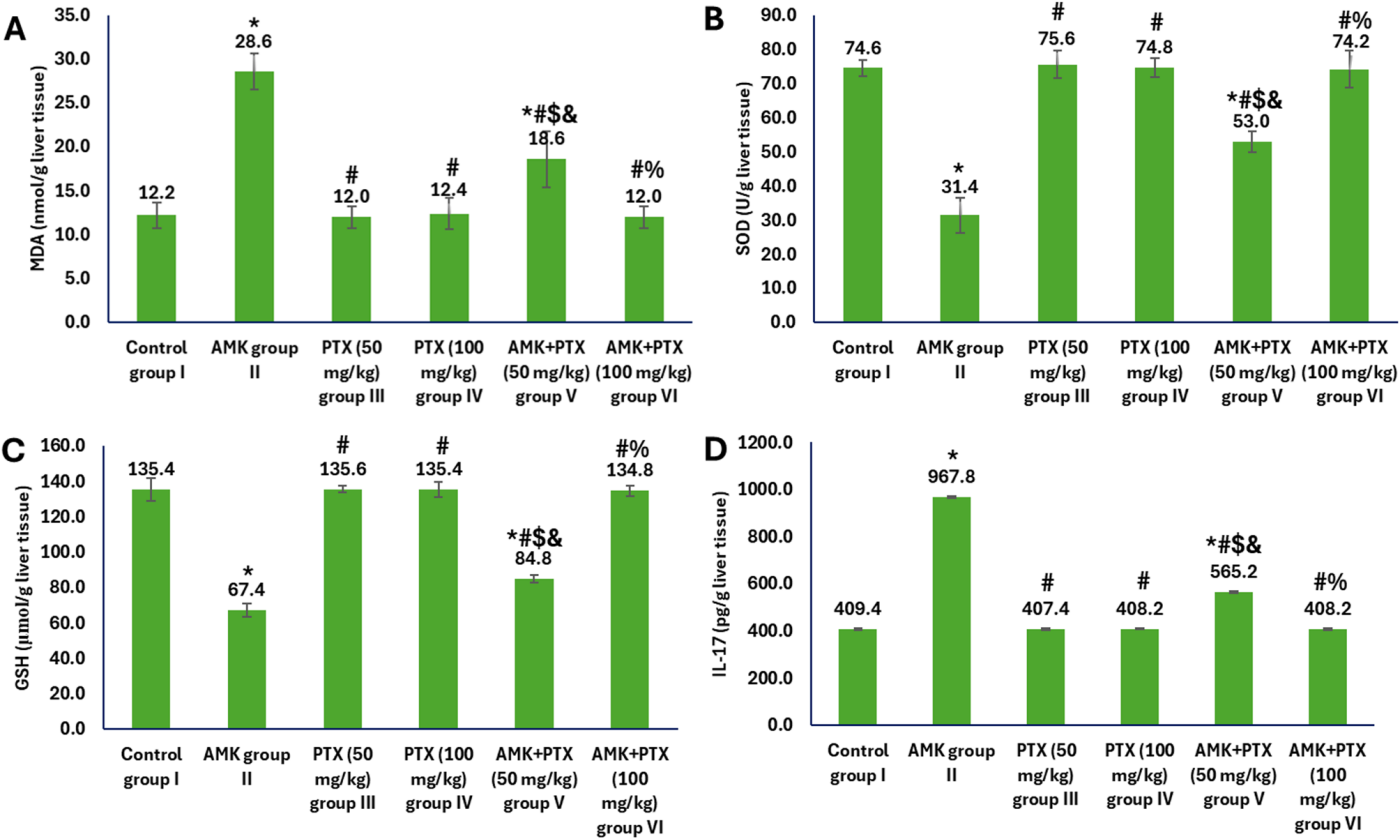
oxidative stress and inflammatory markers in different experimental groups showing levels of MDA (**A**), SOD (**B**), GSH (**C**) and IL-17 (**D**) in liver tissue homogenates. ^*, #, $, & and %^ represented significance (*p* < 0.05) with respect to control group I, AMK group II, PTX (50 mg/kg) group III, PTX (100 mg/kg) group IV and AMK + PTX (50 mg/kg) group V

Moreover, the evaluation of oxidative stress and inflammatory markers in liver tissue demonstrated significant differences among treatment groups (Fig. 4). In group II, administrated with AMK, we observed a substantial increase in MDA levels (28.6 nmol/g liver tissue) compared to controls (12.2 nmol/g liver tissue), indicating severe lipid peroxidation and oxidative damage. This was accompanied by a marked depletion in the antioxidant defence system, with SOD activity reduced to 31.4 U/g liver tissue (versus 74.6 U/g liver tissue in control group I) and GSH level declining to 67.4 µmol/g liver tissue (versus 135.4 µmol/g liver tissue in control group I). The AMK administrated group II also showed a pronounced inflammatory response, with IL-17 levels elevated to 967.8 pg/g liver tissue compared to 409.4 pg/g liver tissue in control group I. PTX monotherapy at both 50 mg/kg (group III) and 100 mg/kg (group IV) maintained all measured parameters at levels comparable to the control group, confirming the absence of hepatic toxicity from PTX administration alone. The combination of AMK with the lower PTX dose (50 mg/kg, group V) showed partial hepatoprotection, significantly attenuating but not completely preventing AMK-induced changes. Most notably, co-administration of AMK with the higher PTX dose (100 mg/kg, group VI) completely prevented AMK-induced damage, with all markers restored to control levels (MDA: 12.0 nmol/g liver tissue; SOD: 74.2 U/g liver tissue; GSH: 134.8 µmol/g liver tissue; IL-17: 408.2 pg/g liver tissue).

### Histopathological examination

**Fig. 5:**
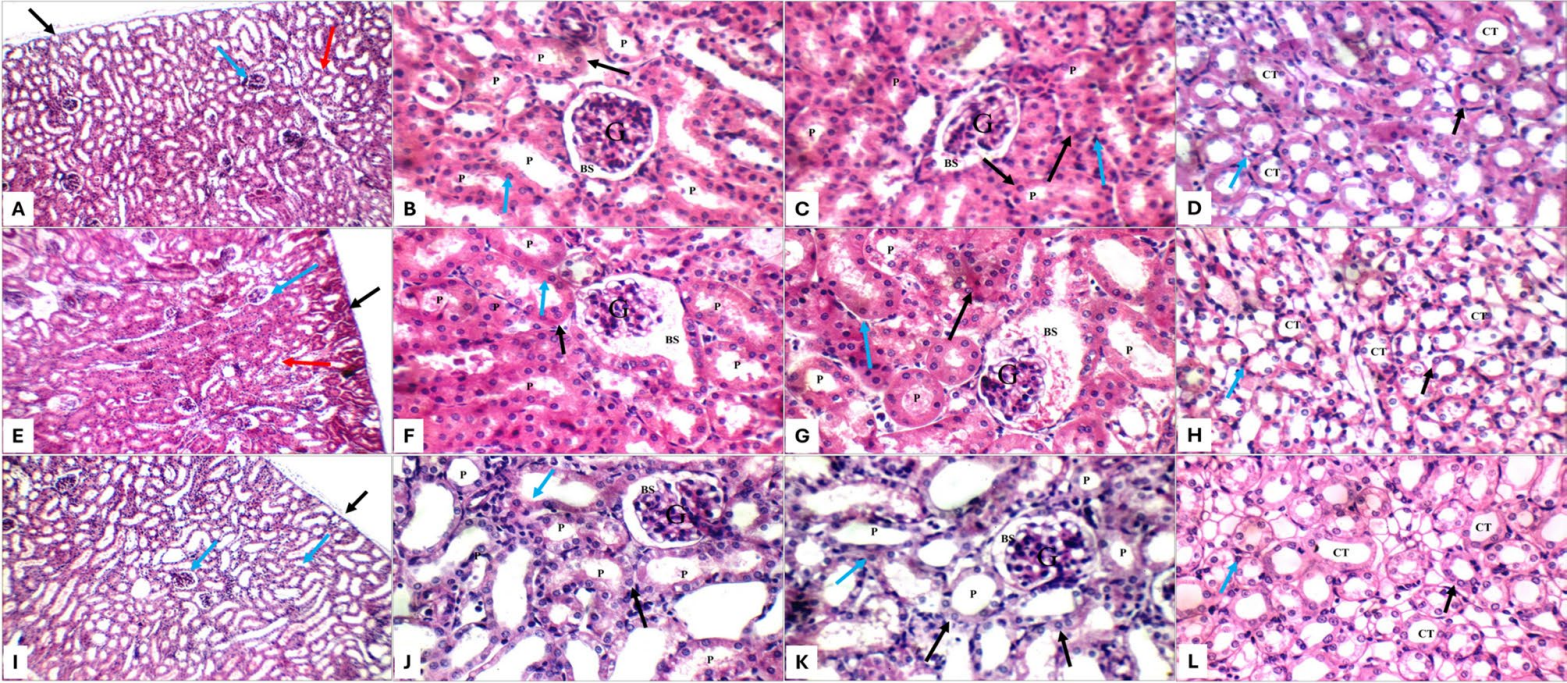
Kidney histopathology (H&E staining) for: Control group I (**A**–**D**) showing normal renal architecture: intact capsule (black arrow), glomeruli (blue arrow), and tubules (red arrow) at 200× (**A**); preserved glomeruli (G) with Bowman’s spaces (BS), proximal tubules (P) with intact epithelium (black arrow) and brush borders (blue arrow) at 400× (**B**,** C**); normal collecting tubules (CT) with average epithelial lining (black arrow) and average interstitium (blue arrow) (**D**) AMK-treated group II (**E**–**H**) demonstrating average renal capsule (black arrow), scattered atrophied glomeruli (blue arrow), and average tubules (red arrow) at 200× (**E**); proximal tubules (P) with markedly apoptotic epithelial lining (black arrow), and partial loss of brush borders (blue arrow) at 400× (**F**,** G**); collecting tubules (CT) with markedly edematous epithelial lining (black arrow) and average interstitium (blue arrow) at 400× (**H**) PTX (50 mg/kg) group III (I-L) showing average renal capsule (black arrow), average glomeruli (blue arrow), and average tubules at 200x (**I**); proximal tubules (P) with average epithelial lining (black arrow), and preserved brush borders (blue arrow) at 400× (**J**,** K**); collecting tubules (CT) with average epithelial lining (black arrow) and average interstitium (blue arrow) at 400× (L)

**Fig. 6:**
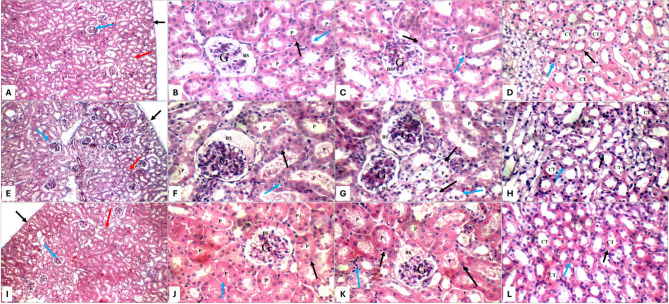
Kidney histopathology (H&E staining) for: PTX (100 mg/kg) group IV (**A**–**D**) showing average renal capsule (black arrow), average glomeruli (blue arrow), and average tubules (red arrow) at 200× (**A**); proximal tubules (P) with average epithelial lining (black arrow), and preserved brush borders (blue arrow) at 400× (**B**,** C**); normal collecting tubules (CT) with average epithelial lining (black arrow) and average interstitium (blue arrow) at 400× (**D**) AMK-PTX (50 mg/kg) group V (**E**–**H**) demonstrating average renal capsule (black arrow), scattered atrophied glomeruli (blue arrow), and average tubules (red arrow) at 200× (**E**); atrophied glomeruli (**G**) with widened Bowman’s spaces (BS), proximal tubules (P) with markedly apoptotic epithelial lining (black arrow), and partial loss of brush borders (blue arrow) at 400× (**F**,** G**); collecting tubules (CT) with edematous epithelial lining (black arrow) and average interstitium (blue arrow) at 400× (**H**) PTX (100 mg/kg) group VI (**I**–**L**) showing average renal capsule (black arrow), average glomeruli (blue arrow), and average tubules (red arrow) at 200x (**I**); proximal tubules (P) with average epithelial lining (black arrow), and preserved brush borders (blue arrow) at 400× (**J**,** K**); collecting tubules (CT) with average epithelial lining (black arrow) and average interstitium (blue arrow) at 400× (**L**)

**Fig. 7:**
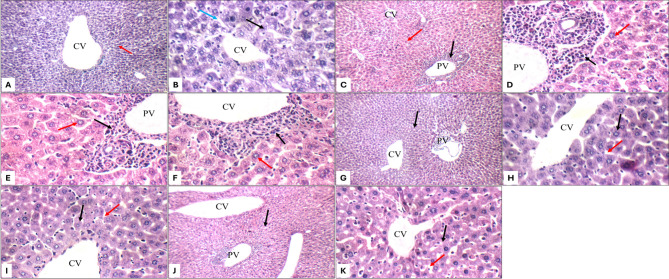
Liver histopathology (H&E staining) for: Control group I (A-B) showing average central vein (CV) and average hepatocytes (red arrow) at 200× (**A**); average hepatocytes arranged in single-cell cords (black arrow) with average blood sinusoids (blue arrow) at 400× (**B**) AMK-treated group II (**C**–**F**) portal tracts with moderate portal inflammatory infiltrate (black arrow), average portal vein (PV), average central veins (CV), and average hepatocytes (red arrow) at 200× (**C**); portal tracts with moderate portal inflammatory infiltrate (black arrow), average portal vein (PV), and average hepatocytes in peri-portal area (red arrow) at 400× (**D**,** E**); average central vein (CV) with mild peri-venular inflammatory infiltrate (black arrow), and average hepatocytes in peri-venular area (red arrow) at 400× (**F**) PTX (50 mg/kg) group III (**G**–**I**) showing average hepatocytes (black arrow) at 200x (**G**); average hepatocytes arranged in single-cell cords (black arrow) with average blood sinusoids (red arrow) at 400× (**H**,** I**) PTX (100 mg/kg) group IV (**J**–**K**) showing average hepatocytes (black arrow) at 200x (**J**); average hepatocytes arranged in single-cell cords (black arrow) with average blood sinusoids (red arrow) at 400× (**K**)

Histopathological examination of kidney and liver sections showed distinct morphological changes among treatment groups. Control animals exhibited normal renal architecture, characterized by intact renal capsules, well-preserved glomeruli, and healthy tubules (Fig. 5A–D). They also showed normal hepatic architecture (Fig. 6), characterized by well-organized portal tracts, intact central veins, and healthy hepatocytes arranged in characteristic single-cell cords with preserved sinusoidal spaces (Fig. 7A, B). AMK-treated animals (group II) showed significant histopathological alterations, including glomerular atrophy and tubular damage. Atrophied glomeruli exhibited widened Bowman’s spaces, while proximal tubules displayed apoptotic epithelial cells and partial brush border loss (Fig. 5E–H). Significant hepatic alterations, including moderate portal inflammatory infiltrates (Fig. 7C, D) and mild peri-venular inflammation (Fig. 7F) were observed in AMK-treated animals. While hepatocytes in both peri-portal (Fig. 7D, E) and peri-venular areas (Fig. 7F) appeared largely normal, the presence of inflammatory cells indicated early hepatic injury. Both PTX monotherapy groups (groups III and IV) maintained normal renal histology comparable to control group I, with preserved glomerular and tubular structures at all magnifications (Figs. 5I–L and 6A–D). A completely normal liver histology, mirroring control specimens was also observed (Fig. 7G–K).

**Fig. 8:**
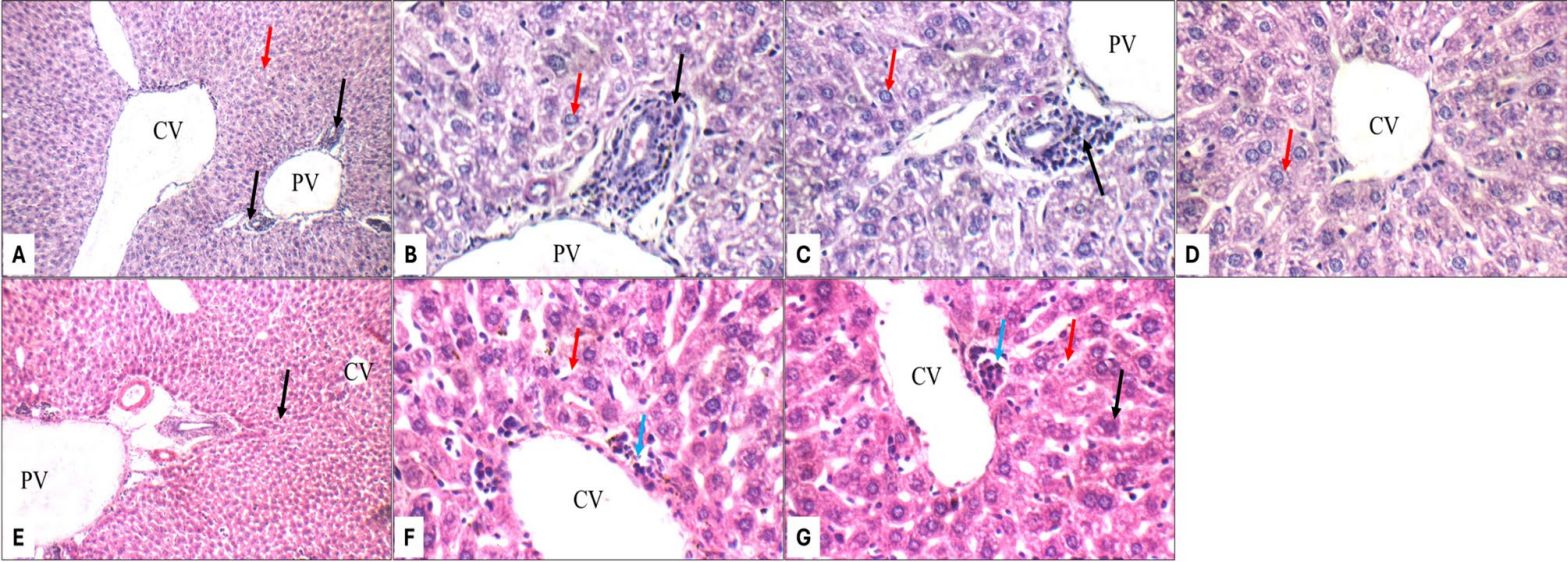
Liver histopathology (H&E staining) for: AMK-PTX (50 mg/kg) group V (**A**–**D**) showing portal tracts with mild portal inflammatory infiltrate (black arrow), mildly dilated portal vein (PV), markedly dilated central veins (CV), and average hepatocytes (red arrow) at 200× (**A**); mild portal inflammatory infiltrate (black arrow), mildly dilated portal vein (PV), and average hepatocytes in peri-portal area (red arrow) at 400× (**B**,** C**); average hepatocytes in peri-venular area (black arrow) at 400× (**D**) AMK-PTX (100 mg/kg) group VI (**E**–**G**) showing average portal tracts (black arrow), mildly dilated portal vein (PV) and average central veins (CV) (red arrow) at 200× (**E**); little peri-venular inflammatory infiltrate (blue arrow), average hepatocytes in peri-venular area (black arrow) and average blood sinusoids (red arrow) at 400× (**F**,** G**)

The combination treatment groups revealed dose-dependent protection patterns. The AMK + PTX (50 mg/kg) co-treatment group V showed partial protection, with scattered glomerular atrophy (Fig. 6E) and persistent tubular epithelial apoptosis (Fig. 6F, G), though less severe than AMK-positive group II. Moreover, a mild portal inflammation (Fig. 8A, B), vascular changes including mildly dilated portal veins and markedly dilated central veins (Fig. 8A) were observed, though hepatocyte morphology remained normal (Fig. 8B–D). Notably, the AMK + PTX (100 mg/kg) group VI demonstrated complete histological protection, with kidney morphology indistinguishable from controls (Fig. 6I–L). This group exhibited near-complete liver histological protection, with only minimal peri-venular inflammation (Fig. 8F) and otherwise normal hepatic architecture, including average portal tracts, central veins, hepatocytes, and blood sinusoids (Fig. 8E–G).

## Discussion

AMK therapy carries significant risks of systemic toxicity that require careful clinical consideration. The antibiotic’s most well-documented adverse effect is dose-dependent nephrotoxicity, characterized by renal tubular damage and impaired kidney function that may lead to fluid retention, electrolyte imbalance, and increased infection risk (Chan et al. 2020). Ototoxicity represents another major concern, with AMK potentially causing irreversible vestibular and cochlear damage manifesting as tinnitus, hearing loss, or vertigo—effects shown to be more pronounced with prolonged treatment durations (Lerner et al. 1986). Neurotoxicity may also occur through disruption of neuromuscular transmission, resulting in muscle weakness, numbness, or seizures, particularly in patients with pre-existing conditions like myasthenia gravis (Lacy et al. 1993). Moreover, hepatotoxicity was linked to AMK administration in animal and human studies. The AMK-induced hepatotoxicity is characterized by oxidative stress, mitochondrial dysfunction, and histopathological damage in liver. Studies demonstrated elevated liver enzymes, increased lipid peroxidation, and reduced antioxidant enzymes levels following AMK administration (Azirak and Özgöçmen 2023). Clinically, AMK poses significant safety constraints: it is absolutely contraindicated in pregnancy due to fetal ototoxicity (Pacifici and Marchini 2020) and exhibits hazardous interactions with nephrotoxic (cisplatin, loop diuretics) or incompatible (penicillins) drugs (Sales and Foresto 2020). Rare but severe hypersensitivity reactions further underscore the need for cautious use (Aronson 2009). Consequently, AMK is reserved for life-threatening Gram-negative infections, mandating rigorous therapeutic drug monitoring, including serum level assessments and hepatic/renal function tests, to mitigate risks (Kovačević et al. 2016).

With increasing antibiotic resistance and few alternatives for severe infections, developing protective agents, such as antioxidants or anti-inflammatory drugs, is crucial to enhance AMK’s safety profile. A protective drug could reduce oxidative stress, prevent cellular apoptosis, and allow for optimal dosing without organ damage, thereby improving treatment outcomes and expanding therapeutic options. Such an approach would preserve AMK’s efficacy while minimizing toxicity-related complications in clinical practice.

This study investigated whether PTX could protect against kidney and liver damage caused by the antibiotic AMK in mice. The study examined PTX’s effects through multiple approaches: measuring blood markers of organ function, assessing oxidative stress levels (MDA, SOD and GSH), analyzing inflammatory responses (IL-17), and evaluating tissue structure under microscopy. IL-17 is a pro-inflammatory cytokine primarily produced by γδ T cells and Th17 cells (Beringer and Miossec 2018) that plays a pivotal role in drug-induced organ injury. While originally studied in autoimmune diseases, IL-17 has emerged as a key mediator in toxicological responses, including acetaminophen-induced hepatotoxicity (Zhu and Uetrecht 2013; Li et al. 2010a) and aminoglycoside nephrotoxicity (Martinez Valenzuela et al. 2020). Mechanistically, IL-17 amplifies tissue damage through multiple pathways: (1) recruiting neutrophils and macrophages to injured sites (Li et al. 2010b), (2) synergizing with reactive oxygen species to exacerbate oxidative stress (Paintlia et al. 2011), and (3) promoting apoptosis through caspase-3 activation (Bockerstett et al. 2018). In the context of AMK toxicity, we selected IL-17 as our primary inflammatory marker because: (1) IL-17 secretion induces an increase in fibrosis of renal tissues (Weng et al. 2020), (2) IL-17 levels correlate strongly with histopathological damage scores in drug-induced nephrotoxicity (Chan et al. 2014), and (3) PTX has demonstrated specific IL-17 suppression in multiple organ injury models (Maldonado et al. 2020). The protective effects of IL-17 neutralization in acetaminophen models further support its central role in xenobiotic-induced injury (Zhang et al. 2023). While other cytokines (e.g., TNF-α, IL-6) certainly contribute to AMK toxicity, IL-17 represents a convergence point for both inflammatory and oxidative pathways, making it particularly relevant for evaluating PTX’s pleiotropic protective effects.

Aminoglycosides, though primarily nephrotoxic, can induce hepatic oxidative stress due to ROS generation (Aşçı et al. 2015; Mehboob et al. 2021). The study by Azirak and Özgöçmen (2023) showed that AMK administration induced significant hepatotoxicity, evidenced by elevated liver enzymes, increased oxidative stress, and upregulated pro-apoptotic markers (Caspase-3 and TNF-α). In the present study, AMK administration induced severe renal dysfunction, marked by significant elevations in BUN, creatinine, uric acid, and albumin levels that was consistent with previous reports of aminoglycoside-induced nephrotoxicity (Karasawa and Steyger 2011; Aşçı et al. 2015). These biochemical alterations were accompanied by substantial oxidative stress in kidney tissues, as indicated by elevated MDA levels and depleted SOD and GSH activities, alongside increased IL-17, suggesting AMK-induced renal injury was mediated, at least in part, by oxidative and inflammatory pathways (Chan et al. 2020). Similarly, AMK-induced hepatotoxicity was characterized by elevated ALT, AST, and ALP serum levels; increased oxidative stress and inflammation in liver tissue. These results were in agreements with Habeeb (2023) who demonstrated that AMK (30 mg/kg/day for 28 days) significantly elevated liver enzymes (AST, ALT, CPK) and oxidative stress markers (MDA) in adult male rats, indicating hepatotoxicity. Compared to the control group, AMK-treated rats showed 3–5-fold increases in ALT and AST levels, suggesting hepatocellular damage.

Moreover, the histopathological examination of kidney and liver sections revealed huge alterations that supported the nephrotoxic and hepatotoxic effects of AMK observed in this study. Martines et al. (1988) administered 40 mg/kg of AMK twice a day for a week and observed ultrastructural liver damage, including primary and secondary microcholestasis (evidenced by thickened pericanalicular webs, biliary microthrombi, and disrupted mitochondrial cristae) and mild phospholipidosis (lipid droplet accumulation). Biochemically, they reported the increased level of BUN and AST, suggesting mitochondrial damage. The study concluded that AMK, though less hepatotoxic than other aminoglycosides, still poses risks, particularly in patients with preexisting liver disease, due to its biliary secretion and tissue accumulation. Cha et al. (2008) investigated the impact of liver disease on the nephrotoxic effects of AMK. They conducted a retrospective chart review of 90 patients who received at least three doses of AMK between December 2005 and August 2006. The researchers concluded that AMK should be used cautiously in liver disease patients, particularly those with hypoalbuminemia. The study by Kadam et al. (2013) investigated the histopathological damage caused by AMK in albino rats. The researchers administered daily doses of AMK for 3, 6, and 9 days and observed progressive damage to liver and kidney tissues. In the liver, AMK caused hepatocyte degeneration, disruption of hepatic cords, widening of bile canaliculi, and reduced Kupffer cells. In the kidney, it led to glomerular collapse, Bowman’s capsule disintegration, and tubular damage, including swelling of proximal convoluted tubules and vacuolation. These effects were dose- and time-dependent, with severe structural breakdown after 9 days, impairing organ function and potentially leading to death. The study highlighted the cytotoxic effects of AMK, emphasizing its risks to hepatic and renal tissues.

In this study, PTX co-administration, particularly at 100 mg/kg, remarkably normalized renal function markers and restored oxidative and inflammatory balance. These findings aligned with prior studies highlighting PTX’s antioxidant and anti-inflammatory properties (Zhang et al. 2005; Garcia et al. 2015). The histopathological results further corroborated these biochemical findings, with AMK causing glomerular atrophy, tubular apoptosis, and brush border loss, while PTX preserved renal architecture. These observations were consistent with reports that PTX mitigated injuries by reducing apoptosis and maintaining structural integrity (Dhulqarnain et al. 2021; Wang et al. 2024; Alherz et al. 2025).

The current study demonstrated that PTX exerted significant dose-dependent protective effects against AMK-induced nephrotoxicity and hepatotoxicity, as evidenced by biochemical, oxidative stress, inflammatory, and histopathological analyses. AMK-induced hepatotoxicity was significantly attenuated by PTX. The hepatic protection was associated with reduced lipid peroxidation (MDA), restored antioxidant defenses (SOD and GSH), and blocked inflammation (IL-17), supporting the idea that PTX’s hepatoprotective effects were mediated through oxidative stress inhibition and cytokine modulation (Zhang et al. 2005; Garcia et al. 2015). Histologically, PTX prevented AMK-induced portal and peri-venular inflammation, further validating its role in preserving hepatic microstructure. The dose-dependent efficacy of PTX suggested its therapeutic potential as an adjuvant during AMK therapy. The complete normalization of renal and hepatic parameters at 100 mg/kg PTX indicated that higher doses may be necessary for optimal protection, a finding supported by previous work demonstrating dose-responsive benefits of PTX in drug-induced organ injury (Dhulqarnain et al. 2021; Wang et al. 2024; Alherz et al. 2025).

The protective effects of PTX observed in this study aligned with its well-documented efficacy across diverse organ toxicity models. Beyond its established benefits in AMK-induced toxicity, PTX has demonstrated significant organ protective potential through multiple mechanisms. For hepatic protection, PTX attenuated acetaminophen-induced hepatotoxicity by reducing oxidative stress and inflammation through NF-κB inhibition (Abdel Salam et al. 2005). Similarly, it protected against D-galactosamine-induced liver injury by suppressing caspase-3 activation (Taye et al. 2009) and recently was shown to ameliorate carbamazepine toxicity via CYP3A4 and NF-κB downregulation (Mohammed et al. 2024). For renal protection, PTX synergized with thiamine to provide superior renal protection through dual inhibition of TLR4/NF-κB signaling and NLRP3 inflammasome-mediated pyroptosis in rhabdomyolysis-induced AKI (Al-Kharashi et al. 2023). This mirrors our findings of PTX’s anti-pyroptotic effects in AMK nephrotoxicity. For cardiopulmonary protection, PTX reduced doxorubicin cardiotoxicity by maintaining antioxidant capacity and preventing mitochondrial dysfunction (Elshazly et al. 2016), while in pulmonary fibrosis models, it decreased collagen deposition by 75% via TGF-β suppression (Entzian et al. 1997). This multi-organ protective profile, combined with its established clinical safety, positions PTX as a unique therapeutic candidate for drug-induced toxicities, particularly in multi-organ injury scenarios.

In conclusion, AMK accumulated in tissues (kidneys and liver) through prolonged exposure and selective uptake by renal proximal tubules and hepatocytes, leading to significant organ damage. AMK-induced toxicity was due to oxidative stress (evidenced by elevated MDA level and depleted antioxidant enzyme levels) and inflammation (marked by upregulated IL-17 level). PTX effectively counteracted AMK-induced nephrotoxicity and hepatotoxicity by preserving organ function, reducing oxidative stress, and suppressing inflammation. These findings advocate for further clinical exploration of PTX as a protective agent in patients requiring aminoglycoside therapy.

While this study demonstrates PTX’s protective effects against AMK-induced toxicity, some limitations should be acknowledged. First, as an animal model, these findings require clinical validation in humans. Second, our 28-days study period focused on acute toxicity rather than chronic effects. Third, while the two PTX doses showed clear dose-dependent protection, additional doses could further optimize therapeutic parameters. Fourth, although IL-17 was selected for its established role in AMK toxicity and PTX’s anti-IL-17 activity, other inflammatory mediators (e.g., TNF-α, IL-6) may contribute and warrant future investigation. Finally, incorporating more sensitive early biomarkers could enhance detection of subclinical toxicity.

## Data Availability

All data generated or analysed during this study are available from the corresponding author on a reasonable request.

## Abbreviations

AMK: Amikacin
PTX: Pentoxifylline
ROS: Reactive oxygen species
MDA: Malondialdehyde
SOD: Superoxide dismutase
GSH: Reduced glutathione
TNF-α: Tumor necrosis factor-alpha
IL-6: Interleukin-6
IL-17: Interleukin-17
ALT: Alanine aminotransferase
AST: Aspartate aminotransferase
ALP: Alkaline phosphatase
BUN: Blood urea nitrogen
AKI: Acute kidney injury
CYP2B1/2: Cytochrome P450 2B1/2 enzymes
ELISA: Enzyme-linked immunosorbent assay
H&E: Hematoxylin and eosin (stain)
IACUC: Institutional animal care and use committee
ARRIVE: Animal research: reporting of in vivo experiments (guidelines)
MIC: Minimal inhibitory concentration
AGE: Advanced glycation end products (related to oxidative stress)
i.p.: Intraperitoneal (administration route)
SD: Standard deviation
ANOVA: Analysis of variance
